# A Comparison of the Anxiolytic Properties of Tofisopam and Diazepam: A Double-Blind, Randomized, Crossover, Placebo-Controlled Pilot Study

**DOI:** 10.3390/ph17010140

**Published:** 2024-01-22

**Authors:** Andrzej Kokoszka

**Affiliations:** II Department of Psychiatry, Medical University of Warsaw, 8 Kondratowicza Street, 03-242 Warsaw, Poland; andrzej.kokoszka@wum.edu.pl

**Keywords:** tofisopam, benzodiazepines, anxiolytics, homophtalazines, diazepam

## Abstract

New clinical reports have recently been published on tofisopam—an anxiolytic drug currently registered as a benzodiazepine—after a long break in this research area. Neurobiological studies concerning its properties, which differ from those of benzodiazepines, are underway. The analyses presented in this study aimed to compare the effects of tofisopam, diazepam, and a placebo in the treatment of anxiety symptoms. A total of 66 outpatients (43 women and 23 men) with generalized anxiety disorder aged 19 to 74 years (M = 41.4; SD = 13.2) were randomized in three groups receiving (1) tofisopam (50 mg three times a day), (2) diazepam (5 mg three times a day), or (3) a placebo for 2 weeks. Then, throughout a 2-week washout period, the patients were monitored for withdrawal symptoms. During the last 2 weeks, the effects of tofisopam (50 mg three times a day) and diazepam (5 mg three times a day) were compared (crossover design). The mean improvement on the Hamilton Anxiety Rating Scale was significantly higher in both the tofisopam and diazepam groups compared to the placebo group. There were no significant differences between the effects of diazepam and tofisopam, whereas adverse effects and withdrawal symptoms occurred less frequently in the tofisopam group. Tofisopam did not impair cognitive abilities, and related withdrawal symptoms resembled those of the placebo. If larger future studies corroborate these findings, tofisopam should be classified as a homophtalazine.

## 1. Introduction

Tofisopam belongs to the class of 2,3-benzodiazepines. It has been approved and used as one of numerous benzodiazepines in several countries, including France, Hungary, Lithuania, Latvia, the Czech Republic, and Slovakia in the European Union, as well as Japan, Argentina (since the 1970s), and recently, India [1]. Some neurobiological data suggest that 2,3-benzodiazepines (in animal studies) and tofisopam (in clinical research) may have different properties from classic 1,4-benzodiazepines. Eventually, it was proposed that 2,3-benzodiazepines should be considered homophtalazines, a new class of compounds. Although their mechanisms of action remain unclear, they have different properties from 1,4-benzodiazepines. The results of the animal studies suggest that the anxiolytic properties of 2,3-beznodiazepines, including tofisopam, are related to their specific binding to the basal ganglia [2]. Differences between homophtalazines and 1,4-benzodiazepines were observed in some early clinical studies. Tofisopam was marketed in Japan as a medication “improving the balance of the autonomic nervous system”. Currently, it is registered as a benzodiazepine.

Bond and Lader [3] reported that tofisopam had no sedative effect and was a slight stimulant. It appeared that tofisopam did not cause any cognitive impairment and even improved performance. Furthermore, no interactions with ethanol were proven [4,5,6,7,8,9]. Despite the data indicating that tofisopam may essentially be different from other benzodiazepines and cause an anxiolytic effect without the risk of dependency and sedation, it was eventually registered as a benzodiazepine. In addition, due to the fact that it has long been put into use, it is now a low cost drug. This may discourage expensive clinical trials that are congruent with the current standards of evidence-based medicine. However, it remains under-investigated, although some researchers are continuing to conduct investigations into it. The D-enantiomer of tofisopam (dextofisopam) is currently being investigated in phase II trials in the United States for the treatment of irritable bowel syndrome.

Recently, new clinical reports on tofisopam have been published. In the context of the renewed interest in research on this drug, the results of a previously unpublished analysis are valuable, as there have only been a few studies on this drug that used randomization and a placebo. The studys was conducted in 2001, according to what is now considered an outdated treatment paradigm for f generalized anxiety disorder (GAD), as part of a drug registration process that was eventually not completed. However, it may shed light on new data and encourage further research.

The presented analysis focuses on the comparison of the impact of tofisopam, diazepam, and a placebo on anxiety symptoms in patients with GAD. It also compares adverse effects and withdrawal symptoms related to the use of the studied drugs.

### 1.1. Theoretical Framework

The main goal of previous studies on the subject was to achieve a preliminary verification of the hypothesis that there are neurobiological differences in the mechanisms of action of tofisopam and benzodiazepines. Furthermore, the results, which were relatively low-quality, indicated similar effectiveness that could be confirmed in randomized placebo-controlled trials. Eventually, the profile of action on anxiety disorder symptoms was compared to check if differences in the neurobiological mechanisms of action of those drugs manifest according to varying levels of effectiveness in the amelioration of specific symptoms of anxiety disorders (neuroses according to ICD-10). Differences in side effects and adverse effects were also investigated.

#### 1.1.1. Previous Neurobiological Findings

The distinction between the neurobiological properties of 2,3-benzodiazepines and 1,4-benzodiazepines was supported by the results of animal studies. The key findings indicated the following points:

– Tofisopam does not exert a direct effect on central benzodiazepine receptors in vitro and in vivo [10,11];
– A benzodiazepine receptor antagonist failed to counteract the effects of tofisopam in vivo [12];
– Tofisopam enhanced the binding of classic benzodiazepines [13];
– The binding sites of 2,3-benzodiazepines were identified in vitro only in the striato–pallido–nigral system in studies that used radiolabeled 2,3-benzodiazepines and analyzed chemical lesions in that region [2].

#### 1.1.2. Previous Clinical Studies

A review of the literature demonstrated that 2,3-benzodiazepines only cause slight sedation or no sedation at all, in contrast to classic benzodiazepines [14]. However, there is a gap between neurobiological and clinical studies on tofisopam. Most of these studies were conducted in the 1970s, when tofisopam was approved for use in the countries listed above, and the classification of mental disorders and research methodologies were at lower stages of development than they are currently.

There is only one multicenter, randomized, double-blind study of tofisopam (150 mg/day) versus hydroxyzine (75 mg/day) based in Poland, which was conducted in 2002, including 51 patients with GAD [15]. In that study, similar improvements in anxiety symptoms were assessed using the Hamilton Anxiety Rating Scale (HARS) [16] and reported across both study groups after 2-week-long and 6-week-long treatments. No significant differences were found between the results from the tofisopam group and the historical data of 33 patients treated with diazepam, which was derived from a similar randomized controlled trial (RCT) [15]. There were no significant differences in the frequency of adverse events and the dropout rates in the three study groups. However, the authors did not consider the placebo effect, which is common in the studied population. Moreover, hydroxyzine is a less effective anxiolytic than classic benzodiazepines.

Several years later, the results of a clinical study on dextofisopam were published [17]. That double-blind, placebo-controlled, randomized study on the use of dextofisopam in 140 patients with irritable bowel syndrome showed that the agent’s effect was superior to that of placebo regarding the primary endpoint (*p* = 0.033). Dextofisopam and placebo had similar rates and types of adverse events, with more instances of worsened abdominal pain being observed with dextofisopam (12% vs. 4%) and more cases of headache with the placebo (2% vs. 5%).

#### 1.1.3. Recent Clinical Studies

The clinical efficacy of tofisopam was recently investigated in India. A total of 22 out of 30 patients with organic catatonia (71%) responded to treatment with 50 to 100 mg/day of tofisopam [18]. Apart from that, a case series illustrating the therapeutic role of tofisopam in the treatment of post-COVID-19 neuropsychiatric sequelae was recently published [19].

#### 1.1.4. Recent Neurobiological Research

Of note, tofisopam remains a key theme in neurobiological research. The results of recent animal studies indicate that tofisopam selectively blocks phosphodiesterase isoenzymes, is active in the mouse model of negative symptoms of psychosis, and may ameliorate negative symptoms of schizophrenia [20]. It also has antiamnestic effects [21].

## 2. Results

### 2.1. Effectiveness of Tofisopam, Diazepam, and Placebo in Study Phase I

After randomization, there were 21 people in the tofisopam group, 20 in the diazepam group, and 24 in the placebo group. No significant differences were found between the groups in terms of the mean age as well as in the mean level of anxiety at the beginning of the study, measured with the Hamilton Anxiety Rating Scale (HARS) [22] (M = 21.4 in the tofisopam group; M = 23 in the diazepam group; and M = 22.6 in the placebo group).

The mean decrease in anxiety levels was significant in all of the study groups (Table 1). An analysis of variance (ANOVA) indicated significant differences across the groups (F = 7.85; df = 2, *p* < 0.001). The results of the Duncan test revealed no differences between the tofisopam and diazepam groups. However, the mean decrease in the HARS score for both the tofisopam (*p* < 0.001) and diazepam (*p* < 0.001) groups was greater than in the placebo group.

### 2.2. Analysis of Changes in HARS Scores during 2 Weeks of Treatment (Phase I)

In the placebo group, a statistically significant reduction in symptoms was observed only in 6 out of 13 items (see bold in Table 2).

In the tofisopam group, a statistically significant reduction in anxiety symptoms was observed in 11 out of 13 items (see bold in Table 3). Of note, in contrast to the diazepam and placebo groups, the tofisopam group showed improved cognitive abilities.

Diazepam significantly reduced all symptoms (see bold in Table 4), except for the intellectual ones. Additionally, in contrast to tofisopam and placebo, it improved insomnia.

### 2.3. Comparison of Changes on the Clinical Global Impression (CGI)

A statistically significant improvement was observed in all of the study groups in terms of the scores obtained on the “severity of illness” form of the CGI [23] (Table 5). There was a greater positive change in the “global improvement” form of the CGI when the actively treated groups and the placebo group were compared (χ^2^ = 11.59; df = 2; *p* < 0.003 in the Kruskal–Wallis test).

### 2.4. Change of Neurotic Symptom Severity

The mean decrease in the frequency of neurotic symptoms assessed using the S-II questionnaire [24] was greater in the tofisopam group than in the placebo group (M = 87.6 vs. 38.6; *t* = −2.16; *p* < 0.005), and there was no difference between the tofisopam and diazepam groups (M = 87.6 vs. 81.7; *t* = 0.22; *p* > 0.8).

After 2 weeks of treatment with tofisopam, the Wilcoxon test showed significant changes in the following subscales of S-II: dysthymia (*p* < 0.001), anxiety (*p* < 0.002), insomnia (*p* < 0.002), somatic symptoms (*p* < 0.003), cognitive dysfunctions (*p* < 0.003), social dysfunctions (*p* < 0.05), and dissociation (*p* < 0.05).

### 2.5. Effectiveness of Tofisopam and Diazepam in Study Phase III

A significant (*p* < 0.001) improvement in symptoms noted in the HARS after the second 2-week period of treatment was found for both tofisopam and diazepam during the third phase of the study (detailed results are presented in Table 6), which took place after the 2-week washout period, during which withdrawal symptoms were observed. No significant differences were observed between the tofisopam and diazepam groups in the *t* test (*t* = −0.34; df = 57; *p* = 0.87).

Tofisopam and diazepam were similarly effective according to the “severity of illness” form of the CGI (Table 7), and there were no significant differences between them (Z = −0.57 in the Mann–Whitney test; *p* = 0.57).

No significant differences in the “global improvement” form of the CGI were noted between both groups (Z = −0.57; *p* < 0.57 in the Mann–Whitney test).

### 2.6. Withdrawal Symptoms Related to the Use of Tofisopam, Diazepam, and Placebo in Study Phase II

A comprehensive list of possible withdrawal symptoms was used to analyze drug effects in the group of 59 patients (5 dropouts and incomplete data in 2 individuals), including the placebo cohort—22 people; tofisopam cohort—17; and the diazepam cohort—20. 

The following symptoms showed significantly higher mean values in the Mann–Whitney test in the diazepam group than in the tofisopam group: anxiety (14.44 vs. 22.88), tension (15.03 vs. 22.38), agitation (14.06 vs. 23.2), excessive sweating (15.59 vs. 21.9), nausea (16.5 vs. 21.13), irritability (14.24 vs. 23.5), dysphoria (13.56 vs. 23.63), feeling dizzy (14.5 vs. 22.83), weakness (15.29 vs. 22.15), insomnia (14.94 vs. 22.45), and headache (15.18 vs. 22.45). In the tofisopam group, a higher mean value than in the diazepam group was reported only for a single symptom—tinnitus (21.94 vs. 18.5; *p* < 0.05).

Withdrawal symptoms were more frequent among patients who were treated with diazepam. Significant differences were found in the following groups of symptoms: sensory disorders (χ^2^ = 18.0; df = 2; *p* = 0.001); confusion (χ^2^ = 9.6; df = 2; *p* = 0.008); and “individual” symptoms (χ^2^ = 12.5; df = 2; *p* = 0.002). The frequency of withdrawal symptoms in all subgroups of the tofisopam group did not differ from the one observed in the placebo group.

### 2.7. Adverse Reactions

**Figure 1:**
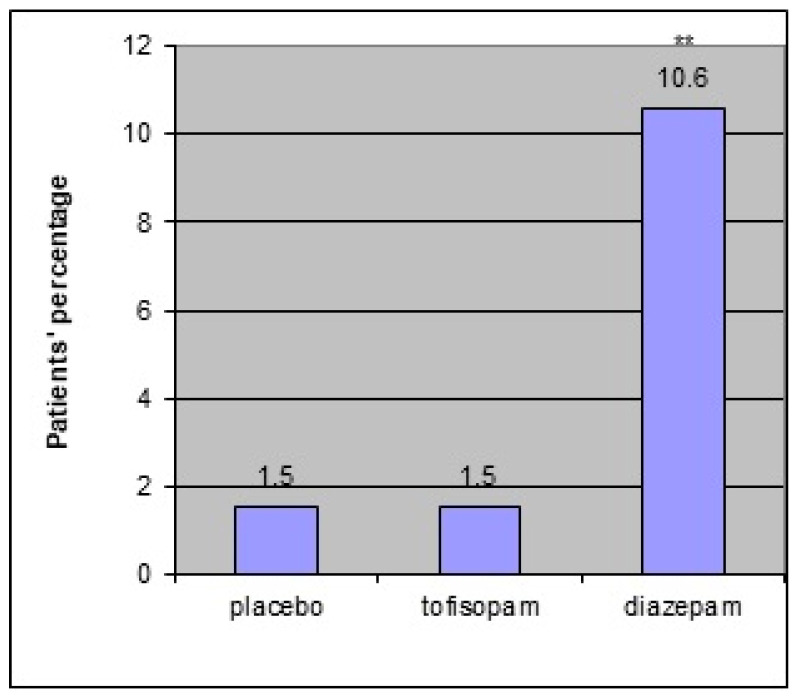
Occurrence of adverse events in study phase I. ** *p* < 0.01 in χ^2^.

There was one serious adverse event during the third phase of the study, namely myocardial infarction. A 51-year-old woman with a history of chronic hypertension suffered a heart attack. However, its relation to the study medication (diazepam in phase III and tofisopam in phase I) was considered to be of low probability. Adverse reactions (see Figure 1 below) were more frequent in the diazepam group (seven people (10.6%)) than in the tofisopam (one person (5.9%)) and placebo (one person (4.3%)) groups (χ^2^ = 11.13, df = 2; *p* < 0.01) in the first phase as well as in the third phase of the study:with fourteen reports (23%) in the diazepam group and one (1.6%) in the tofisopam group (χ^2^ = 12.22, df = 1; *p* < 0.01). 

## 3. Discussion

This study aimed to compare the properties of tofisopam, diazepam, and placebo in the short-term treatment of anxiety. The presence of free-floating anxiety, experienced by patients with GAD for the majority of time, makes this patient group suitable for comparison. This study should not be considered a clinical trial researching the effectiveness and safety of GAD treatment. The results of this study can support hypothesized meaningful differences between tofisopam and diazepam, and probably with other benzodiazepines as well.

One limitation of this study is the small number of participants in the study groups. However, the results indicating a similar global reduction in the symptoms of anxiety in both groups and less sedation, increasing cognitive abilities in tofisopam, and promoting sleep by diazepam encourage further comprehensive studies. Decreased driving abilities are one of the most important adverse effects of classic benzodiazepines. If the lack of impact of tofisopam on the performance of mechanical equipment operators was confirmed, the range of tofisopam applications would significantly increase.

Although there is no significant risk of dependency after 2-week benzodiazepine treatment, the trial was relatively short in duration due to the risk of developing such dependency. However, it was long enough to find many significant differences between tofisopam and diazepam. 

Symptom improvement assessed using the CGI scale was similar for diazepam, tofisopam, and placebo, which may be explained by the relatively low sensitivity of this method. Although tofisopam and diazepam had significantly different profiles of adverse reactions and withdrawal symptoms, both had similar effectiveness in this study. These data support the results obtained from animal studies suggesting that homophtalazines have mechanisms of action that are different from diazepam and that they are very well tolerated and do not cause clinically significant withdrawal symptoms (their profile did not differ from placebo).

The dosages administered in this study may be questioned, as the current guidelines for GAD treatment, e.g., those endorsed by the World Federation of Societies of Biological Psychiatry [25], recommend 5–15 mg a day of diazepam. The dosage of tofisopam was, on average, 3 × 50 mg a day (the highest dose is 3 × 100 mg), whereas the dosage of diazepam was relatively higher than the average, i.e., 3 × 5 mg. This is another limitation of the study, because a relatively higher dosage of diazepam and a longer half-life (24–48 h) than that of tofisopam (6–8 h) may explain the decrease in cognitive abilities on diazepam and the lack of such an impact on tofisopam. 

On the other hand, if the average dosing of tofisopam is as effective as those of relatively high dosages of diazepam, this demonstrates that tofisopam is at least as effective as diazepam or that it may be more effective using higher dosages. Of note, at the end of the 20th century, when the study was designed, a higher dosage of diazepam was recommended, e.g., according to the “Oxford Textbook of Psychiatry”, diazepam was recommended for GAD in dosages ranging from 5 mg twice daily in mild cases to 10 mg three times daily in the most severe cases [26] (pp. 182–183). Therefore, this may explain why 5 mg of diazepam was taken three times daily as an average dosage when the study was carried out.

Due to the small number of study participants, the conclusions from this pilot study are only preliminary. However, the results encourage further research to verify these findings, e.g., in a multicenter RCT, probably with the use of equivalent doses of the study medication.

## 4. Materials and Methods

### 4.1. Study Population

A total of 66 outpatients (43 women and 23 men) with GAD, aged between 19 and 74 years (M = 41.4; SD = 13.2), were enrolled in the study in 2 outpatient clinics in Poland, and the study was completed by 61 participants. There were 4 dropouts during the first phase of the study (2 individuals withdrew informed consent due to personal reasons unrelated to the study, and 2 patients refused to continue participation because of the sedative effects of the drug) and 1 dropout during the third phase, which was due to a serious adverse event not related to the investigated medication (hospitalization). 

### 4.2. Methods

#### 4.2.1. Assessment Methods

Trained psychiatrists were raters in the study and used the following tools:

- The Structured Mini-International Neuropsychiatric Interview (MINI), version 5.0.0 plus [27,28] was used for the comprehensive psychiatric diagnosis.
- The Montgomery–Åsberg Depression Rating Scale (MADRS) [22], a structured interview, was applied for the evaluation of the intensity of depressive symptoms and the exclusion of patients with significant depressive symptoms at the initial assessment.
- The Hamilton Anxiety Rating Scale (HARS) [16], a structured interview, was used to assess the intensity of anxiety symptoms.
- The Clinical Global Impression (CGI) scale [23] was used to evaluate the general severity of symptoms.
- Anxiety symptoms (neurotic according to the ICD-10 still used in Poland) were assessed with the self-rating symptom check list S-II [24], a Polish derivative of the Symptom Checklist-90 (SCL-90) developed by Derogatis, which evaluates patients’ subjective experience of symptoms.
- A comprehensive list of possible withdrawal symptoms, based on a review of the literature, was used for diagnosis. The list includes 34 symptoms classified as mild, moderate, or severe (enclosed in the Supplementary File).

#### 4.2.2. Inclusion Criteria

The inclusion criteria were as follows:

- GAD diagnosed according to the MINI, version 5.0.0.;
- Lack of any other mental disorders, including alcohol and psychoactive substance use disorders, within the previous 6 months;
- Fewer than 21 points on the MADRS;
- Aged 18 years or older;
- No anxiolytic or antidepressant treatment within 7 days prior to the study, and no buspirone therapy within 30 days prior to the study;
- No benzodiazepines detected in the blood.

#### 4.2.3. Exclusion Criteria

The exclusion criteria included serious medical conditions, pregnancy, and breastfeeding.

### 4.3. Study Design

A crossover study design was used, with 3 consecutive 2-week phases (treatment duration was limited due to the risk of developing benzodiazepine dependency):

- Phase I: A central randomization procedure was performed by phone call. All patients were randomized to one of the three groups:
  - Tofisopam (50 mg 3 times a day)—21 people (including 2 dropouts);
  - Diazepam (5 mg 3 times a day)—20 people;
  - Placebo (3 times a day)—25 people (including 2 dropouts).
- Phase II: All patients were monitored for withdrawal symptoms and did not receive any medications during the washout period.
- Phase III: Drug effectiveness was compared between the two study groups:
  - Tofisopam (50 mg 3 times a day)—31 people;
  - Diazepam (5 mg 3 times a day)—29 people (including 1 dropout).

**Scheme 1:**
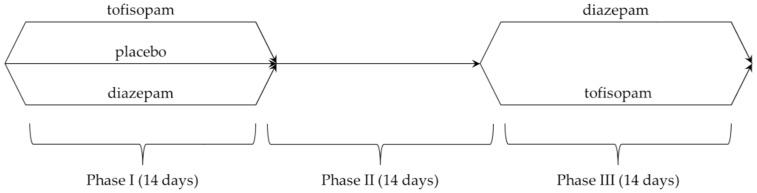
Study design.

Patients participating in the study received identical pills containing 50 mg of tofisopam or 5 mg of either diazepam or placebo. All patients treated with active agents in phase I were switched to another medication; patients from the placebo group were randomized using a central randomization procedure by phone call. The details are presented in Scheme 1.

### 4.4. Ethical Approval

This study was approved by the Bioethics Committee of the Medical University of Warsaw, permission KB/81/K/2001. All patients signed an informed consent form.

### 4.5. Statistical Analysis

All statistical analyses for the clinical trial 0123/EG were performed with SPSS software, version 11.4. A *p* value ≤ 0.05 was considered significant.

An intention-to-treat (ITT) analysis was used to assess the safety of therapy (analysis of adverse events and treatment discontinuation) in all patients who took at least one tablet of the study drug (n = 66). Efficacy analysis was performed in all patients who underwent at least one follow-up examination (n = 62).

Prior to the comparative analyses, the Kolmogorov–Smirnov test was used to verify the hypothesis on the normal distribution of the study groups. Group homogeneity was assessed with the *t* test in study phase I (tofisopam—diazepam—placebo) and phase III (tofisopam—diazepam).

The mean laboratory test results in particular groups among women and men were compared with an analysis of variance.

The paired-samples *t* test was used to compare changes in the total score on the HARS between phase I (follow-up visit 1–2) and phase III (follow-up visit 3–4). Its use was justified, as the total HARS score can be treated as a continuous variable, and the Kolmogorov–Smirnov test did not provide evidence to reject the hypothesis on normal data distribution.

The difference in outcomes between phase I (visits 1 and 2) and phase III (visits 3 and 4) was verified with an analysis of variance with the Duncan multiple range test and *t* test for independent samples accordingly, depending on the medication used.

Anxiety decreases related to medications used in phase I were compared with the analysis of variance and the post hoc Duncan test (comparison of 3 mean values), whereas decreases associated with drugs used in phase III were analyzed with the *t* test (comparison of 2 mean values).

## 5. Conclusions

To conclude, this pilot study shows that tofisopam may be as effective as diazepam in the short-term treatment of anxiety symptoms. It had significantly fewer adverse effects and withdrawal symptoms than diazepam. The number of adverse effects related to the use of tofisopam did not differ from those noted in the placebo group. Further RCTs with a larger number of participants are needed to confirm the preliminary findings suggesting that tofisopam has an anxiolytic effect and does not exacerbate cognitive impairment.

## Figures and Tables

**Table 1:** Change in anxiety levels in study phase I according to the results of the Hamilton Anxiety Rating Scale (HARS).

	Study Day 1			Study Day 14			Change	*t* Test
Group	N	Mean	SD	N	Mean	SD		
Placebo	23	22.57	4.05	23	19.70	4.55	−2.87	*t* = 3.59; df = 22; *p* < 0.01
Tofisopam	19	21.63	3.45	19	14.00	3.76	−7.63	*t* = 7.18; df = 18; *p* < 0.001
Diazepam	20	23.05	3.58	20	15.95	4.52	−7.20	*t* = 6.94; df = 19; *p* < 0.001

**Table 2:** Change in severity of anxiety symptoms listed on the Hamilton Anxiety Rating Scale (HARS) in the placebo group.

Item	Visit 1				Visit 2				
	Median	Mode	Min	Max	Median	Mode	Min	Max	Wilcoxon Signed-Rank Test
**1. Anxious mood**	**2**	**2**	**2**	**3**	**2**	**2**	**1**	**4**	**Z = −2.00; *p* = 0.046**
**2. Tension**	**3**	**3**	**1**	**3**	**2**	**2**	**1**	**4**	**Z = −2.352; *p* = 0.019**
3. Fears	1	1	0	3	1	1	0	3	Z = −1.897; *p* = 0.058
4. Insomnia	2	2	1	3	2	2	1	3	Z = 0.00; *p* = 1.000
5. Intellectual symptoms	2	2	1	3	2	2	1	3	Z = −1.897; *p* = 0.058
6. Depressed mood	2	2	1	3	2	2	1	3	Z = −1.265; *p*= 0.206
**7. Somatic—general (muscular and sensory)**	**2**	**2**	**1**	**3**	**1**	**1**	**0**	**3**	**Z = −2.333; *p* = 0.020**
8. Cardiovascular symptoms	1	1	0	3	1	1	0	2	Z = −0.447; *p* = 0.655
**9. Respiratory symptoms**	**2**	**2**	**0**	**3**	**1**	**1**	**0**	**2**	**Z = −2.714; *p* = 0.007**
10.Gastrointestinal symptoms	1	1	0	2	1	1	0	2	Z = −0.577; *p* = 0.564
**11. Genitourinary symptoms**	**1**	**1**	**0**	**3**	**1**	**1**	**0**	**3**	**Z = −2.00; *p* = 0.046**
**12. Autonomic symptoms**	**2**	**2**	**0**	**3**	**1**	**1**	**1**	**2**	**Z = −2.333; *p* = 0.020**
13. Behavior at interview	1	1	0	2	1	1	0	3	Z = −1.134; *p* = 0.257

**Table 3:** Change in severity of anxiety symptoms listed on the Hamilton Anxiety Rating Scale (HARS) in the tofisopam group.

Item	Visit 1				Visit 2				
	Median	Mode	Min	Max	Median	Mode	Min	Max	Wilcoxon Signed-Rank Test
**1. Anxious mood**	**2**	**2**	**1**	**3**	**1**	**1**	**0**	**3**	**Z = −3.213; *p* < 0.001**
**2. Tension**	**2**	**2**	**1**	**3**	**1**	**1**	**1**	**2**	**Z = −3.176; *p* < 0.001**
**3. Fears**	**2**	**1**	**1**	**3**	**1**	**1**	**0**	**2**	**Z = −2.840; *p* = 0.005**
**4. Insomnia**	**2**	**2**	**0**	**3**	**1**	**1**	**0**	**2**	**Z = −2.807; *p* = 0.005**
**5. Intellectual symptoms**	**2**	**2**	**1**	**3**	**1**	**2**	**0**	**2**	**Z = −3.357; *p* < 0.001**
**6. Depressed mood**	**2**	**2**	**1**	**3**	**1**	**1**	**0**	**3**	**Z = −2.887; *p* = 0.004**
7. Somatic—general (muscular and sensory)	2	2	0	2	1	1	0	2	Z = −1.933; *p* = 0.053
**8. Cardiovascular symptoms**	**2**	**2**	**0**	**3**	**1**	**1**	**0**	**2**	**Z = −3.464; *p* < 0.001**
**9. Respiratory symptoms**	**2**	**2**	**1**	**3**	**1**	**1**	**0**	**2**	**Z = −3.207; *p* < 0.001**
**10.Gastrointestinal symptoms**	**1**	**1**	**0**	**3**	**1**	**1**	**0**	**2**	**Z = −2.070; *p* = 0.038**
11. Genitourinary symptoms	1	1	0	3	1	1	0	2	Z = −1.667; *p* = 0.096
**12. Autonomic symptoms**	**2**	**2**	**0**	**2**	**1**	**1**	**0**	**2**	**Z = −3.162; *p* = 0.002**
**13. Behavior at interview**	**1**	**1**	**0**	**3**	**1**	**0**	**0**	**3**	**Z = −3.127; *p* = 0.002**

**Table 4:** Change in severity of anxiety symptoms listed on the Hamilton Anxiety Rating Scale (HARS) in the diazepam group.

Item	Visit 1				Visit 2				
	Median	Mode	Min	Max	Median	Mode	Min	Max	Wilcoxon Signed-Rank Test
**1. Anxious mood**	**3**	**3**	**2**	**3**	**2**	**2**	**1**	**2**	**Z = −3.755; *p* < 0.001**
**2. Tension**	**2**	**2**	**2**	**3**	**1**	**1**	**1**	**3**	**Z = −3.704; *p* < 0.001**
**3. Fears**	**2**	**1**	**1**	**3**	**1**	**1**	**0**	**3**	**Z = −2.887; *p* = 0.004**
**4. Insomnia**	**2**	**2**	**0**	**3**	**1**	**2**	**0**	**3**	**Z = −2.810; *p* = 0.005**
5. Intellectual symptoms	2	2	1	3	2	2	0	3	Z = −1.414; *p* = 0.157
**6. Depressed mood**	**2**	**2**	**1**	**3**	**1**	**1**	**1**	**3**	**Z = −2.530; *p* = 0.011**
**7. Somatic—general (muscular and sensory)**	**2**	**1**	**1**	**3**	**1**	**1**	**0**	**2**	**Z = −2.828; *p* = 0.005**
**8. Cardiovascular symptoms**	**2**	**2**	**0**	**3**	**1**	**1**	**0**	**3**	**Z = −3.771; *p* < 0.001**
**9. Respiratory symptoms**	**2**	**2**	**0**	**3**	**1**	**1**	**0**	**2**	**Z = −2.887; *p* = 0.004**
**10.Gastrointestinal symptoms**	**1**	**1**	**1**	**3**	**1**	**1**	**0**	**3**	**Z = −2.449; *p* = 0.014**
**11. Genitourinary symptoms**	**1**	**1**	**0**	**2**	**1**	**1**	**0**	**2**	**Z = −2.309; *p* = 0.021**
**12. Autonomic symptoms**	**2**	**2**	**1**	**2**	**1**	**1**	**0**	**2**	**Z = −3.000; *p* = 0.003**
**13. Behavior at interview**	**1**	**1**	**1**	**3**	**1**	**0**	**0**	**3**	**Z = −2.673; *p* = 0.008**

**Table 5:** Results of the “severity of illness” form of the Clinical Global Impression (CGI) scale in study phase I.

	Study Day 1					Study Day 14					
Group	N	Median	Mode	Min	Max	N	Median	Mode	Min	Max	Wilcoxon Test
Placebo	23	4	3	3	6	23	3	3	1	6	Z = −3.116; *p* = 0.002
Tofisopam	17	4	4	3	1	17	3	3	1	4	Z = −3.256; *p* = 0.001
Diazepam	20	5	5	3	6	20	3	2	1	5	Z = −3.621; *p* < 0.001

**Table 6:** Results of Hamilton Anxiety Rating (HARS) in study phase III.

	Study Day 1			Study Day 14			Change	*t* Test
Group	N	Mean	SD	N	Mean	SD		
Tofisopam	31	19.7	4.3	31	14.1	3.9	−5.62	*t* = 11.11; df = 30; *p* < 0.001
Diazepam	28	18.4	4.7	28	12.8	4.6	−5.64	*t* = 7.51; df = 27; *p* < 0.001
All	59	19.1	4.5	59	13.5	4.3	−5.62	

**Table 7:** Results of the “severity of illness” form of the Clinical Global Impression (CGI) scale in study phase III.

	Study Day 1					Study Day 14					
Group	N	Median	Mode	Min	Max	N	Median	Mode	Min	Max	Wilcoxon Test
Tofisopam	31	4	4	2	5	31	3	3	1	5	Z = −4.399; *p* < 0.001
Diazepam	28	4	4	1	5	28	3	3	1	4	Z = −4.055; *p* < 0.001I

## Data Availability

The data belong to Egis Pharmaceuticals. Some data were presented as posters at congresses and their abstracts were published [29,30].

## Supplementary Materials

The following supporting information can be downloaded at: https://www.mdpi.com/article/10.3390/ph17010140/s1, A list of withdrawal symptoms.
