# Supplementary material for: A Comparison of the Anxiolytic Properties of Tofisopam and Diazepam: A Double-Blind, Randomized, Crossover, Placebo-Controlled Pilot Study
Source: Pharmaceuticals (Basel). 2024 Jan 22;17(1):140. doi: 10.3390/ph17010140 (PMC10820453; doi:10.3390/ph17010140)
Supplement: Supplementary file 1 [file pharmaceuticals-17-00140-s001.zip › pharmaceuticals-2772609-supplementary.pdf]

LIST OF WITHDRAWAL SYMPTOMS

GROUP	SYMPTOM	SEVERITY			
		0 – none	1 – mild	2 – moderate	3 –severe
**I Adrenergic**	Anxiety				
	Restlessness				
	Psychomotor agitation				
	Loss of appetite				
	Excessive sweating				
	Nausea				
	Vomiting				
**II Lethargy**	Annoyance				
	Tiredness				
	Loss of libido				
	Lethargy				
	Dysphoria				
	Constipation				
**III Sensory disorders**	Feeling dizzy				
	Loss of balance				
	Movement coordination disorders				
	Tinnitus				
	Perceptual (sensory) disorders				
**IV Confusion**	Increased sensitivity to sounds and smells				
	Difficulty with concentrating				
	Depersonalization				
	Difficulty with expressing thoughts				
	Confusion				
	Nightmares				
**V Neurasthenia**	Weakness				
	Tremor in the limbs				
**VI Muscular symptoms**	Muscle cramps				
	Fasciculation				
**VII Individual**	Insomnia				
	Headaches				
	Diarrhea				
	Other psychotic reactions				
	Hallucinations				
	Seizures				

SYMPTOM SEVERITY SCALE

0 – none; 1 – mild; 2 – moderate; 3 – severe
